# The Embryonic Chick Femur Organotypic Model as a Tool to Analyze the Angiotensin II Axis on Bone Tissue

**DOI:** 10.3390/ph14050469

**Published:** 2021-05-16

**Authors:** Thais Francini Garbieri, Victor Martin, Carlos Ferreira Santos, Pedro de Sousa Gomes, Maria Helena Fernandes

**Affiliations:** 1 Department of Biological Sciences, Bauru School of Dentistry, University of São Paulo, Bauru, São Paulo 17012-901, Brazil; tfgarbieri@usp.br (T.F.G.); cfsantos@fob.usp.br (C.F.S.); 2 Laboratory for Bone Metabolism and Regeneration, Faculty of Dental Medicine, University of Porto, 4200-393 Porto, Portugal; up201908935@edu.fmd.up.pt (V.M.); pgomes@fmd.up.pt (P.d.S.G.); 3 LAQV/REQUIMTE, University of Porto, 4160-007 Porto, Portugal

**Keywords:** embryonic chick femur organotypic model, AngII/AT1R/AT2R axis, bone formation, bone deterioration, angiotensin II, AT1 and AT2 receptors, receptor blockade

## Abstract

Activation of renin–angiotensin system (RAS) plays a role in bone deterioration associated with bone metabolic disorders, via increased Angiotensin II (AngII) targeting Angiotensin II type 1 receptor/Angiotensin II type 2 receptor (AT1R/AT2R). Despite the wide data availability, the RAS role remains controversial. This study analyzes the feasibility of using the embryonic chick femur organotypic model to address AngII/AT1R/AT2R axis in bone, which is an application not yet considered. Embryonic day-11 femurs were cultured ex vivo for 11 days in three settings: basal conditions, exposure to AngII, and modulation of AngII effects by prior receptor blockade, i.e., AT1R, AT2R, and AT1R + AT2R. Tissue response was evaluated by combining µCT and histological analysis. Basal-cultured femurs expressed components of RAS, namely ACE, AT1R, AT2R, and MasR (qPCR analysis). Bone formation occurred in the diaphyseal region in all conditions. In basal-cultured femurs, AT1R blocking increased Bone Surface/Bone Volume (BS/BV), whereas Bone Volume/Tissue Volume (BV/TV) decreased with AT2R or AT1R + AT2R blockade. Exposure to AngII greatly decreased BV/TV compared to basal conditions. Receptor blockade prior to AngII addition prevented this effect, i.e., AT1R blockade induced BV/TV, whereas blocking AT2R caused lower BV/TV increase but greater BS/BV; AT1R + AT2R blockade also improved BV/TV. Concluding, the embryonic chick femur model was sensitive to three relevant RAS research setups, proving its usefulness to address AngII/AT1R/AT2R axis in bone both in basal and activated conditions.

## 1. Introduction

The endocrine renin–angiotensin system (RAS) has a key role in the control of blood pressure, blood volume, and fluid balance, and its activation participates in the development and/or progression of cardiovascular, renal, and metabolic diseases [1]. In this system, angiotensinogen (AGT) synthesized and released from the liver is converted to angiotensin I (AngI) by renin, which is released from the juxtaglomerular cells of the kidney [2]. The angiotensin-converting enzyme (ACE) activates AngI to AngII, which attains high levels on the vasculature endothelial cell surface [2]. AngII, the most dynamic component of RAS, acts via Angiotensin type 1 and Angiotensin type 2 G-protein-coupled receptors (R) that exhibit ≈34% amino acid sequence identity [2]. Long-term activation of ACE/AngII/AT1R is mostly associated with harmful effects, whereas the majority of studies support the notion that AT2R may mediate opposite effects being mainly involved in protective mechanisms [1]. Otherwise, the RAS branch mediated by Ang1-7, formed by the cleavage of AngII by ACE2, appears to have modulatory effects on the AngII-mediated RAS by acting on the G protein-coupled receptor Mas (MasR) attenuating AT1R-mediated negative effects [1,2,3,4]. In addition to the endocrine RAS, local/tissue RAS exerting autocrine and paracrine effects modulate the activity of multiple tissues and organs, including the bone tissue [5,6], playing an important role in physiological and pathological conditions. Thus, this system has a role in the cellular fate, namely cell migration, proliferation, differentiation, and apoptosis [2]. Otherwise, alterations on its function appears relevant in several diseases [2].

The biological relevance of systemic and local RAS seems significant in conditions of its activation. Regarding the bone tissue, available clinical, in vivo, and cellular data strongly suggest that RAS activation plays a role in bone deterioration associated with metabolic disorders such as osteoporosis, arthritis, bone, risk fracture, and fracture healing, due to the increased activity of AngII acting via AT1R [6,7,8,9]. In line with this, therapeutic approaches involving RAS inhibition, such as the use of Angiotensin I converting enzyme inhibitors (ACEI) and Angiotensin II type 1 receptor blockers, appears to have positive bone effects by adjusting the balance of AngII [10,11,12].

A variety of clinical and meta-analytical studies involving the use of ACE inhibitors (ACEI) and AngII receptor blockers (ARBs, namely AT1R blockers) in the treatment of conditions such as hypertension, cardiac failure, and diabetic nephropathy suggest beneficial effects in the bone mineral density and risk of fractures [10,12,13,14,15]. However, a recent meta-analysis found that the association of ACEI and ARBs with decreased risk of bone fractures remains inconsistent [16]. Additionally, RAS modulation with AngII, ACEI, and ARBs (type 1 and type 2 receptors) assessed in experimental disease models of osteoporosis, spontaneous and induced hypertension, diabetes, and knockout models [8,10,17,18,19] showed both positive results [20,21] or no effect [22,23]. Great variability is also reported in cell culture models with diverse degrees of complexity and involving a multiplicity of experimental protocols of RAS stimulation/inhibition, trying to elucidate the subjacent cellular and molecular mechanisms [8,10,12,21,24]. Nevertheless, available data converge to the notion that the osteoblasts appear to be the major targets underlying the negative effects of AngII in bone metabolic activity and the increased osteoclastic activity seen in conditions of RAS activation, which appear to be an indirect osteoblast-mediated effect [8,10,25,26,27]. 

Despite the wide variety of studies, the role of RAS activation and modulation remain controversial, with several reasons accounting for this, namely related to the clinical setting, concerns arising from the quality of protocols and the risk of bias in studies involving animal models [28,29], and poor correlation between in vitro and in vivo studies addressing bone regeneration [30]. 

In this context, ex vivo systems emerge as useful tools with several advantages, namely the preservation of the 3D tissue cellular environment [31]. The embryonic chick limb organotypic model has been used in bone-related biology to address development, regeneration, and responses to external stimuli [32,33]. A major advantage of this model is the rapid development of the skeleton within the 3D cellular/matrix setup, as in vivo, which is essential for the phenotype differentiation and maintenance of the differentiated cells involved in tissue formation [32,33]. Furthermore, the availability of the genome sequence [34] allows mechanistic studies. In this model, 11th day (ED11) embryonic femurs are removed and cultured ex vivo in the air/medium interface during 10–11 days. At ED11, the immature femur is rich in undifferentiated progenitor cells; thus, it is highly responsive to external stimuli being a useful tool for biological manipulation [32]. This embryonic day is considered the optimal point to establish the organotypic femur culture because skeletal differentiation has just started and the diaphysis bone collar is formed [32]. However, vascular infiltration has not yet occurred, limiting the presence of immune cells and osteoclastic cells by this stage [32]. During the ex vivo culture, after a period of approximately two days for femurs recovery from the dissection procedure, a stage of cell proliferation and steady growth of bone and cartilage occurs in the following 3–12 days [32]. The difference in the development and growth between avian and human bone is a major limitation; however, it appears that the cellular and signaling events are similar across both species [32]. Furthermore, the absence of immune system and vascularization limits its representativeness, although these features might be attractive in some research settings [32,33]. Overall, the model remains useful, cheap, and easy to implement and manipulate, bridging the gap between cell culture systems and in vivo models while fulfilling the 3Rs of reduction, refinement, and replacement and has been used in a number of bone-related applications from bone development biology, testing of biomaterials and drugs, and tissue regeneration and engineering strategies [35,36,37,38]. 

In addition to the advantages inherent to an ex vivo model, the organotypic culture of embryonic chick femur presents some features that seem particularly appellative to address the bone RAS. Thus, the absence of an immune system and the lack of vascularization during the ex vivo bone formation [32] avoids the complex immune/bone tissue interactions [39] and the effect of systemic RAS on local bone-associated RAS [2], respectively. Furthermore, as osteoclasts are mostly absent, the model allows directing the RAS role and modulation to the bone formation, which is an interesting issue considering that osteoblastic-lineage cells are the main suggested bone cell type targeted by RAS [10,25,26,27].

Considering the above observations, this study aims to analyze the feasibility of the ex vivo embryonic chick femur model to address the AngII/AT1R/AT2R axis and the possibility of its modulation, which is an application tool that has not yet been investigated. First, the gene expression of RAS components was confirmed for ACE (angiotensin-converting enzyme), Ang II, AT1, AT2, and Mas receptors. Then, the model was set up in order to characterize the system in basal conditions, its responsiveness to exogenous AngII aiming its activation, and the possibility of mitigating AngII effects by receptor blockade (AT1R; AT2R; AT1R + AT2R), anticipating a suitable tool to screen potentially useful drugs. Tissue response was assessed by combining microcomputed tomography (µCT) and histological characterization.

## 2. Results

The ex vivo embryonic chick femur model was set up using the experimental protocol previously optimized and described [32,33]. Eleven-day embryonic femurs were cultured ex vivo for 11 days in the medium/air interface, to increase oxygen tension within the tissue promoting higher viability. Femurs were cultured in the absence of FCS to avoid ectopic mineralization, migration of cells out of the whole femur onto the culture surface and, importantly, to ensure the chemical definition of the culture medium due to FCS batch variability [33]. According to the previous studies, this culture period is the most appropriate to analyze the bone formation, because progressive tissue deterioration occurs for longer culture times [33]. In addition, at ED11, femurs are programmed to drive a bone formation process rather than a bone remodeling process [32].

**Figure 1:**
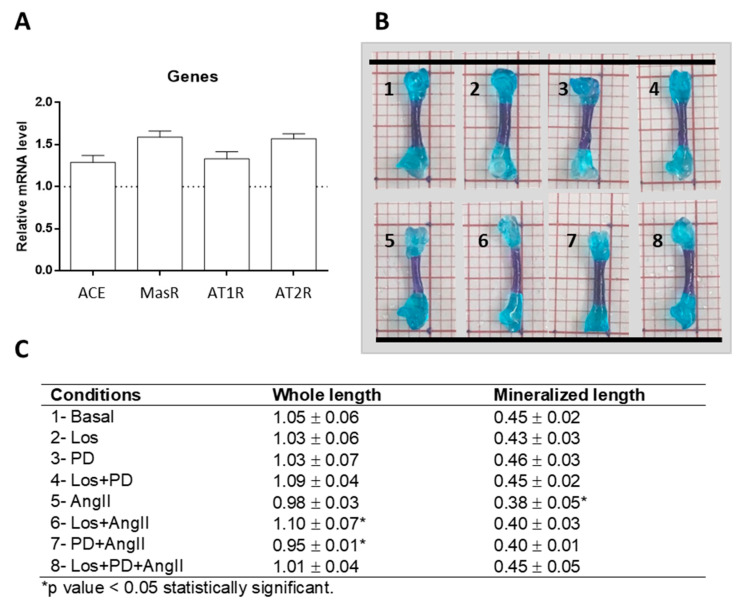
Gene expression (qPCR) of ACE, MasR, AT1R and AT2R of ED11 chick femurs following 11-day organotypic culture in basal conditions (**A**). Whole-mount bone and cartilage staining of chick embryos with Alcian blue and Alizarin red S (**B**). Length of the all femur and diaphyseal mineralized region (**C**).

ED11 femurs cultured ex vivo in basal conditions for 11 days were analyzed for the presence of genes coding for RAS components, as this model has not been previously searched for this application. Results showed that the cultured femurs expressed the genes for ACE, AngII receptors AT1 and AT2, and Mas receptor, as shown in Figure 1A. 

Following, ED11 femurs were grown in basal conditions or treated with AngII (10^−6^ M). In both situations, bone tissue response was modulated by Losartan (Los, 10^−6^ M, AT1R blocker), PD123319 (PD, 10^−5^ M AT2R blocker), or Losartan + PD123319. Concentrations of AngII, Losartan, and PD123319 used in this study have been previously optimized in bone cell culture studies addressing RAS modulation, being routinely used in the in vitro models [20].

As a first approach, at the end of the ex vivo culture, tissue response was checked in the whole femur double-stained with Alcian blue and Alizarin red S, as shown in Figure 1B. The femurs preserved the morphological characteristics and integrity in all tested conditions, and the diaphysis showed a bluish-red staining, suggesting the formation of bone-related tissue in this region. Femurs measured ≈10 mm length and, compared to basal conditions (1.05 ± 0.06 mm), treatment with Los + AngII or PD + AngII resulted respectively in a slight (but significant, *p* ≤ 0.05) increase (1.10 ± 0.07 mm) or decrease (0.95 ± 0.01 mm). The length of the bluish-red stained diaphyseal region was ≈4 mm, and a decrease was observed in the AngII-treated femurs (≈16%, *p* < 0.05). Given the promising results of this preliminary study, the model was further evaluated in more detail aiming to accomplish the proposed objectives.

### 2.1. Microcomputed Tomography 

**Figure 2:**
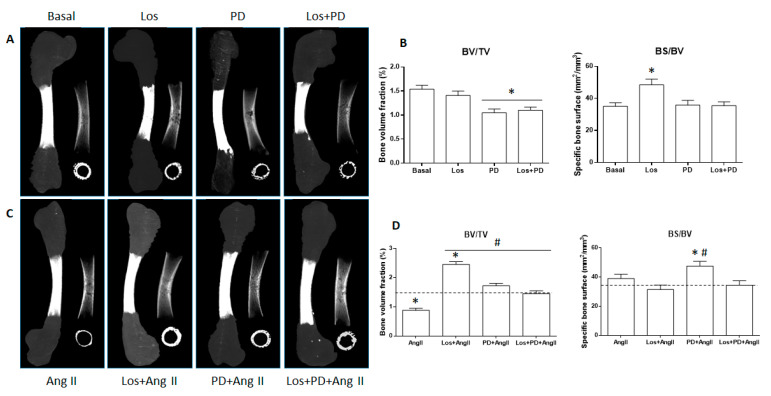
Microcomputed tomography images of the whole femur tissue region (segmented mineralized bone, sagittal and cross-sections of the central diaphysis region) and morphometric indices (BV/TV and BS/BV) of ED11 chick femurs following 11-day organotypic culture in conditions of AngII/AT1R/AT2R axis modulation. (**A**,**B**): basal conditions, and following receptor blockade; (**C**,**D**): exposure to AngII, and receptor blockade prior to the addition of AngII. Los: Losartan; PD: PD123319. * Statistically different from basal conditions; ^#^ statistically different from AngII; (*p* ≤ 0.05).

Figure 2A,B show, respectively, the microcomputed tomography (µCT) images (whole mineralized bone and cross-sectional sections of the central diaphysis) and the morphometric indices (BV/TV; BS/BV) of the femurs cultured in basal conditions and in the presence of Losartan, PD123319, or Losartan + PD123319. µCT images, Figure 2A, particularly the cross-sectional ones, suggest a decrease in the mineralized volume of the diaphyseal region upon AT2R (PD123319, 10^−5^ M) or AT1R + AT2R (Losartan 10^−6^ M + PD123319, 10^−5^ M) blockade. The morphometric indices, as shown in Figure 2B, provided similar information. Basal cultured femurs presented bone volume fraction (BV/TV) values of 1.54% ± 0.08. Upon AT1R blockade, values were similar, whereas blocking AT2R or AT1R + AT2R elicited ≈25 to 30% decrease (*p* < 0.05). The bone surface fraction (BS/BV) displayed a different behavior. Values of 35.1 mm^2^/mm^3^ ± 2.2 in basal conditions increased significantly under the effect of the AT1R blocker Losartan (48.45 mm^2^/mm^3^ ± 3.6; ≈38% increase; *p* < 0.05), but they were similar following the blockade of AT2R or AT1R + AT2R.

ED11 femurs were cultured ex vivo in the presence of AngII (10^−6^ M, 11 days). Compared to the basal cultured femurs, treatment with AngII caused a deleterious effect in bone tissue that was clearly noted in the cross-sectional section of the central diaphysis, as shown in Figure 2C. Accordingly, quantitative analyses showed a significant decrease in the bone volume fraction (BV/TV), ≈45% compared to the control femurs, although bone surface fraction (BS/BV) remained similar, as shown in Figure 2D. 

Subsequently, the effect of AngII was modulated by blocking its receptors. In these experiments, the receptor blocker was added to the medium before the addition of AngII. Results for the µCT data analysis are presented in Figure 2C,D. Losartan (AT1R blocker) completely prevented the negative effects of AngII as suggested in the cross-sectional image and confirmed in the quantification of the bone volume fraction. Thus, BV/TV values increased from 0.88% ± 0.07 in AngII-treated condition to 2.45% ± 0.1 under AT1R blockade (almost three-fold); furthermore, this value was ≈60% higher than that observed in basal conditions (1.54% ± 0.08). Simultaneously, the bone surface fraction (BS/BV) showed a slight decrease, ≈15%. The blockade of AT2R with PD123319 also prevented the negative effects of AngII in bone volume fraction, but values were only slightly higher (1.72% ± 0.09) than those found in basal conditions (1.54% ± 0.08). However, the bone surface fraction was clearly increased, i.e., 47.5 mm^2^/mm^3^ ± 3.2 compared to 35.1 mm^2^/mm^3^ ± 2.1 in the basal-cultured femurs and 38.95 mm^2^/mm^3^ ± 2.97 in AngII-treated femurs. The simultaneous blockade of AT1R and AT2R also avoided the negative effects of AngII, and values for BV/TV and BS/BV were similar to those in basal conditions. 

### 2.2. Histological Evaluation

**Figure 3:**
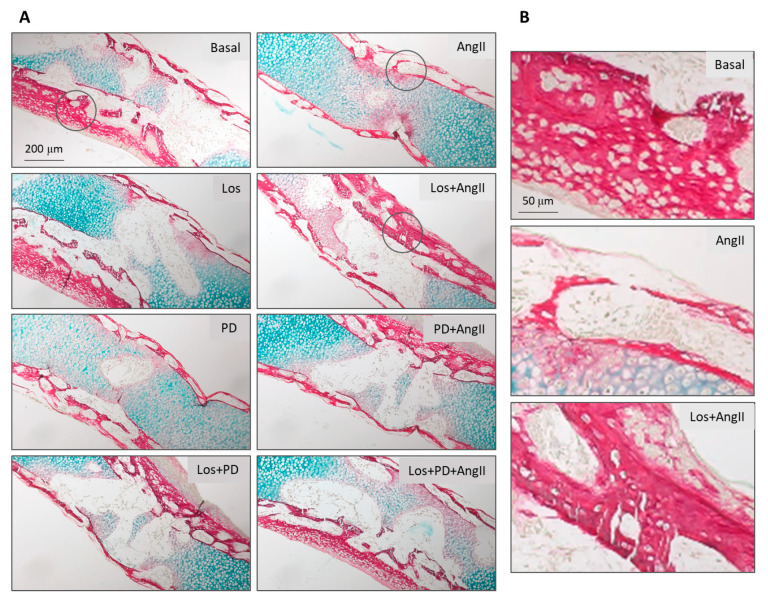
Histological images of the diaphyseal region from ED11 chick femurs stained with Alcian blue and Picrosirius red for glycosaminoglycan and collagenous matrix visualization, following 11-day organotypic culture in conditions of AngII/AT1R/AT2R axis modulation. **A**: Low magnification images illustrating all tested conditions; **B**: high magnification images for basal conditions, exposure to AngII and AT1R blockade prior to the addition of AngII. Los: Losartan; PD: PD123319. Scale bar = 200 µm (**A**) and 50 µm (**B**).

**Figure 4:**
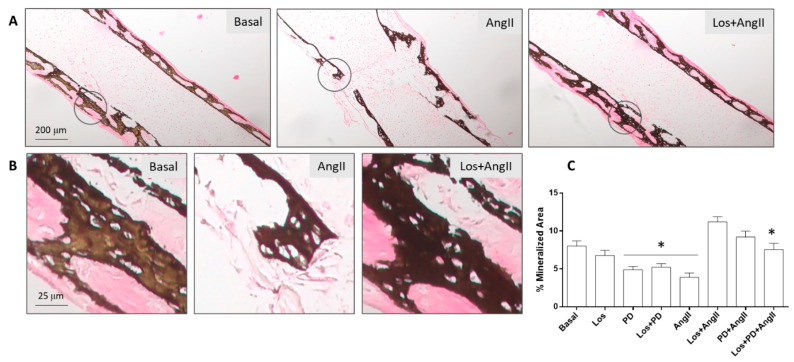
Histological analysis of the diaphyseal region from ED11 chick femurs stained for von Kossa (mineralized collagenous matrix) following 11-day organotypic culture in conditions of AngII/AT1R/AT2R axis modulation. **A**,**B**: Representative images for basal conditions, exposure to AngII and AT1R blockade prior to the addition of AngII; **C**: quantitative assessment of the percentage of mineralized content in all testing conditions. Los: Losartan; PD: PD123319. Scale bar = 200 µm (**A**) and 25 µm (**B**); * statistically different from basal conditions; *p* ≤ 0.05.

The embryonic femurs grown in all conditions were characterized by histochemical staining, with a focused evaluation on femoral diaphysis. Tissue sections were stained with Alcian blue for proteoglycan-rich cartilage matrix and Sirius red for collagenous matrix, as shown in Figure 3. Von Kossa staining was used to evaluate the presence of a mineralized collagenous matrix, as shown in Figure 4.

In basal conditions, tissue sections presented the deposition of a collagenous matrix at the periosteal regions progressing to fill the diaphysis, as seen by the Sirius red staining. This matrix displayed a continuous appearance in the external part of the diaphysis and a trabecular-like organization as it filled this region. However, the inner part of the diaphysis still shows the bluish staining of the proteoglycan-rich cartilage matrix. The diaphysis sections of the femurs treated with AT1R blocker (Losartan) looked enlarged, but the red-colored tissue appeared similar, exhibiting the characteristic trabecular organization, as shown in Figure 3A. Femurs treated with AT2R blocker (PD123319) or AT1R + AT2R blockers appeared with lower amount of collagenous matrix showing some structural disruption, as shown in Figure 3A. 

The presence of AngII (10^−6^ M) during the femur’s ex vivo growth (11 days) caused an evident deleterious effect on the deposition of the collagenous matrix, with a marked decrease in the red-stained areas and in the trabecular-like arrangement, as seen in Figure 3A. 

Blockade of AT1R with Losartan (10^−6^ M) 2 h prior to the addition of AngII had a significant effect (*p* ≤ 0.05) on this behavior. Tissue samples revealed increased collagen matrix deposition with thicker and more developed trabecular organization. The AT2R blockade (PD123319, 10^−5^ M) also modulates the effect of added AngII. Increased matrix deposition was noted, although this effect appeared less pronounced compared to that resulting from the AT1R blockade. In addition, the simultaneous blockade of AT1R and AT2R before the treatment with AngII prevented the drastic negative effect of AngII in bone formation, and the tissue appearance approaches that observed in basal conditions. These results are illustrated in Figure 3A,B.

Von Kossa staining of the tissue sections was positive in all conditions, showing that the mineralized areas matched the red-stained collagenous matrix, and they clearly evidenced the trabecular-like structure. Figure 4 shows representative images for the basal-cultured femurs and those treated with AngII (10^−6^ M) or with Losartan (10^−6^ M) + AngII. The marked negative effect of AngII in the amount of mineral content and trabecular-like organization is evident compared to the basal-cultured femurs. Furthermore, the positive effect of AT1R blockade prior to adding AngII is clearly illustrated. Results for the quantification of the percentage of mineralized area are in line with that observed with the Sirius red staining. In basal conditions, the blockade of AT2R or AT1R + AT2R caused a decrease in the percentage of the mineralized area. Treatment with AngII resulted in a ≈50% reduction, which was prevented by receptor blockade. This effect was more significant with AT1R blockade compared to the other blocking conditions.

## 3. Discussion

First, qPCR analysis proved that ED11 femurs cultured for 11 days expressed components of RAS, namely ACE, AT1R, AT2R, and MasR (Figure 1A). At this stage, the presence of these genes suggested the eventual sensitivity of the model to address the AngII/AT1R/AT2R axis, and no other genes were analyzed. In a preliminary experiment (Figure 1B), ED11 femurs cultured in basal conditions or treated with the modulatory drugs (AngII and receptor blockers) maintained the integrity during the ex vivo culture.

Femurs cultured for 11 days in basal conditions duplicate the behavior described previously [32,33]. 

Together, results suggest that the two AngII receptors mediate different effects on bone formation regarding bone volume fraction and bone surface fraction. In a condition of AT1R blockade, the AT2R-mediated effects would probably assume an increased relevance. This receptor subtype is essentially associated with bone protective effects [6,21,40,41], which might explain the negligible effect in bone volume fraction, although the increased bone surface fraction is suggestive of alterations in the structural features of the formed bone. On the other hand, upon AT2R blockade, AngII-AT1R-mediated effects are prone to predominate, possibly explaining the decreased bone volume fraction, which is in line with the deleterious effects associated to AT1R [6,24], although maintaining the bone surface fraction. Alternatively, the simultaneous blockade of AT1 and AT2 receptors, by preventing AngII effects, opens the possibility of activation or increased relevance of other/alternative RAS axis triggered by feedback regulatory mechanisms [42]. One hypothesis would be the activation of the Ang1-7/MasR axis, i.e., the conversion of AngII into Ang1-7 that, by acting on the MasR, that is expressed in the ex vivo cultured femurs, is associated with protective effects [3]. Still, bone volume fraction and the percentage of mineralized matrix were lower compared to that on basal conditions. Indeed, with AT1R or AT2R blockade, contributions of other RAS pathways might also be hypothesized as a result of the disturbed AngII/AT1R/AT2R axis. Taken together, in the embryonic chick femur model cultured in basal conditions, the role of the AngII/AT1R/AT2R axis seems to prevail over other alternative/triggered RAS pathways. The blockade of AT1R, AT2R, or both receptors caused alterations in bone formation, either in the structural features or the bone volume fraction, and these were not compensated by the eventual triggered feedback RAS regulatory mechanisms. Regarding this, it should be noted that in normal conditions, bone metabolic activity is regulated by major hormonal and local pathways [43], and the relevance of systemic/local RAS is mostly associated, and clinically significant, in conditions of its activation occurring in certain systemic long-term pathologies [2,7,9].

As referred above, the disturbance of bone metabolic activities occurring in a variety of chronic diseases is associated with decreased bone mass and a higher incidence of bone fracture [6,7,8,9]. Within the complexity of the subjacent mechanisms, RAS activation occurring in such conditions, due to increased AngII levels, appears to play a role in bone deterioration [9,10,11]. Considering this, the embryonic chick model was analyzed for its responsiveness to added AngII aiming to mimic a condition of RAS activation. Results coming from µCT and histology clearly showed that femurs grown with AngII presented significantly decreased bone volume fraction (BV/TV), percentage of mineralized collagenous matrix, and disrupted trabecular-like arrangements, validating the responsiveness of the model to this RAS mediator. These effects are in line with the known negative effects of AngII from in vitro and in vivo studies and also clinical assessment [9]. 

The blockade of AT1R prevented the negative effects of AngII in the bone parameters and, in addition, further increased bone volume fraction (BV/TV) and the percentage of mineralized collagenous matrix comparatively to those measured in basal conditions. The effect in the bone surface volume was not significant, although a tendency for a decrease was noted. In this experimental setup, the embryonic chick model behaved similarly to most of the available experimental settings and clinical information concerning the positive effect of Losartan and other AT1R blockers [10,44,45]. The blockade of AT1R would prevent the activation of this receptor by the added AngII and, most probably, it directs the mediator to activate AT2R, favoring a positive effect on bone response, as referred above. Interestingly, in the embryonic chick model, the blockade of AT2R also prevented the negative effects of AngII by increasing BV/TV to levels higher than those in basal conditions, in spite of the expected negative AT1R-mediated effects. However, the stimulatory effect was lower than that observed upon AT1R blockade and, most relevant, the bone surface fraction (BS/BV) increased significantly, suggesting that the bone formed upon AT2R blockade presented lower density. The increase in bone mass was also reported in adult mice after AT2R blockade, AT2 knock-out mice, and osteoblastic cell cultures [21]. 

The above results suggest that AT1R and AT2R might modulate bone volume fraction in conditions associated with increased AngII levels, but with distinct effects in the bone structural architecture. Furthermore, as mentioned above, the contribution of other/alternative RAS pathways arising from adaptive regulatory mechanisms to the disturbed AngII/AT1R/AT2R axis might also occur, i.e., the Ang1-7/MasR axis [42]. It is worth stressing that both bone mass and bone structural features are relevant concerning bone mechanical performance and cellular metabolic activities, having different roles in the prevention of bone fractures associated with diseases going along with RAS activation. The two parameters that present non-linear relationships [46] are bound to vary considerably within the wide range of reported experimental and clinical protocols dealing with disturbed RAS function, receptor modulation, and induced adaptive regulatory pathways difficult to identify. Overall, this is most probably a contributing factor to the great variability and inconsistency of reported results and behavior patterns. Nevertheless, the positive bone effects observed in the embryonic chick model upon the blockade of AT1R or AT2R receptors are in line with previous studies [21]. Again, the embryonic chick model appears responsive to RAS modulation.

**Figure 5:**
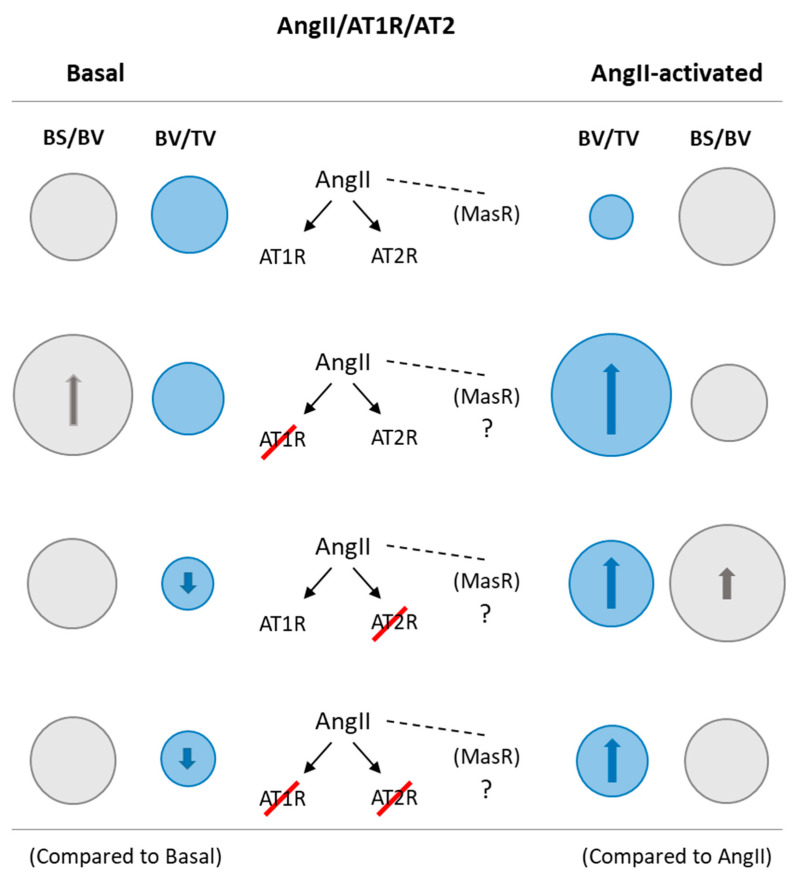
Schematic diagram summarizing the bone responsiveness of ED11 organotypic chick femur model to AngII/AT1R/AT2R axis modulation, for bone volume fraction (BV/TV) and bone surface fraction (BS/BV). Basal: without and with receptor blockade–bone surface fraction increased after AT1R blocking and bone volume fraction decreased with AT2R and AT1R + AT2R blockade. AngII-activated: compared to basal conditions, exposure to AngII greatly decreased bone volume fraction, and receptor blockade prior to the addition of AngII prevented the negative effects of this mediator: AT1R blockade resulted in increased bone volume fraction with maintenance of bone surface fraction, whereas, blocking AT2R caused a lower increase on bone volume fraction and a greater bone surface fraction; AT1R + AT2R blockade also led to increased bone volume fraction.

Figure 5 presents a schematic diagram summarizing the bone response observed after the ex vivo growth of the embryonic chick femur, in terms of bone volume fraction (BV/TV) and bone surface fraction (BS/BV), in conditions of AngII/AT1R/AT2R modulation. The ex vivo bone growth of ED11 chick femurs was sensitive to AngII/AT1R/AT2R modulation, and AT1R and AT2R seem to be differently involved in the elicited bone response. Results are indicative of a major role of AT1R in the AngII negative effects. With the simultaneous blockade of AT1R and AT2R, bone formation was similar to that observed in basal conditions, suggesting the involvement of alternative pathways within RAS axis and/or other bone regulatory mechanisms.

## 4. Materials and Methods

### 4.1. Organotypic Cultures of Embryonic Chick Femurs

Fertilized chick eggs (*Gallus domesticus*) were incubated in an Octagon 40 ECO rotating egg incubator (Brinsea, UK), at 37.5 °C and 50% humidity. At day 11, the embryos were euthanized, and whole femurs were carefully dissected, removing the soft tissue such as ligaments and adherent muscles while preserving the periosteum. Femurs (*n* = 10 per group) were carefully washed in saline and settled onto Netwell™ Insert (440 µm mesh size polyester membrane, 30 mm diameter, Corning) in 6-well tissue culture plates (Costar^®^) containing 1 mL of basal culture medium (1 mL; α-MEM, 100 U/mL penicillin, 100 μg/mL streptomycin, 2.5 μg/mL amphotericin B, 50 μg/mL ascorbic acid 2-phosphate), at the liquid/gas interface, and incubated in a humidified atmosphere of 5% CO_2_/37 °C. After 24 h, the medium was removed, and the embryonic chick femurs were further cultured for 11 days in basal conditions (1 mL of basal medium in the absence of drugs) or treated with AT1 (Losartan, Sigma; 10^−6^ M), AT2 (PD123319, Sigma; 10^−5^ M) receptor blockers or AngII (Sigma; 10^−6^ M). The following conditions were tested: (1) Basal medium, (2) Losartan, (3) PD123319, (4) Losartan + PD123319, (5) AngII, (6) Losartan + AngII, (7) PD123319 + AngII, and (8) Losartan + PD123319 + AngII. In conditions 6 to 8, the receptor blockers were added for 2 h before the addition of AngII. The culture medium was changed every 24 h, and the receptor blockers and/or AngII were present throughout the all culture period. 

At the end of the experiment, cultured femurs were washed in Phosphate-Buffered Saline (PBS, pH = 7.4), fixed, and processed for microtomographic (μCT) and histological analysis, using standardized conditions. In addition, femurs cultured in basal conditions were snap frozen and later processed for gene expression analysis. In addition, whole-mount double staining with Alcian blue and Alizarin red S was performed to visualize the skeletal patterns of the chicken embryo femur.

### 4.2. Whole-Mount Histochemical Femur Staining

Whole-mount double staining with Alcian blue for cartilage and Alizarin red S for bone was performed to allow the visualization of the skeletal patterns of chicken embryo femur in the same specimen, based in a previous work [47]. Briefly, decalcification by the acidic Alcian blue solution (0.01%) was performed overnight (approximately 16 h). After that, a dehydration at 95% ethanol was followed by rehydration in a decreasing graded ethanol series. Then, a staining with Alizarin red S solution (0.002%) in a 0.5% potassium hydroxide for 24 h was followed by an immersion in a KHO solution (2%) for 4 h. Tissue cleaning was performed by immersing the embryos femurs successively in 25%, 50%, and 80% mixtures of 0.5% KOH and glycerin 22h each. Femurs were stored in 100% glycerin prior to imaging on a Zeiss 305 Stereo microscope equipped with a digital camera (Zeiss Axiocam 208). Data analysis was conducted on ImageJ software (version 1.51j8).

### 4.3. Microtomography Evaluation 

Femur specimens were imaged in a SkyScan 1276 micro-computed tomography scanner (Bruker, Kontich, Belgium). Sample containers (1.5 mL Eppendorf tubes) were set on the sample stage and imaged using a detector assembly over a 360° sample rotation. Data were acquired under the following settings: source voltage of 40 kV, source current 100 µA, an exposure time of 800 ms, and a voxel size of 4.5 µm. Raw data were reconstructed in the NRecon software v.1.7.4.2, upon correction for beam hardening, ring artifacts, and misalignment. CT Analyzer software v.1.17.7.2 was used to visualize and analyze the reconstructed images for bone volume (BV), tissue volume (TV), and bone surface (BS). For the histomorphometric analysis, a volume of interest embracing 2 mm in the proximal and distal directions, starting at mid-diaphysis and comprising a total of 900 layers, was defined. Thresholding was applied to obtain an average binarized grayscale for the reconstructed datasets. 

### 4.4. Histological Processing and Histochemical Analysis

Alcian blue and Picrosirius red were combined to produce distinctive staining of collagen (red), proteoglycans (blue), and allow visualizing both collagen and proteoglycan–matrix components on the same histological section, or alternatively, the mineralized tissue upon von Kossa staining [48]. Briefly, cultured femurs, fixed in neutral buffered formalin, were processed for routine paraffin embedding. Sections were deparaffinized, hydrated, and stained in Alcian blue solution, pH 2.5, containing 1g alcian blue (Sigma), 3 mL glacial acetic acid (Fisher), and 97 mL distilled water, for 30 min at room temperature. Afterwards, samples were rinsed in tap water and stained in Picrosirius red solution composed by 0.1 g sirius red (Aldrich) and 100 mL saturated aqueous picric acid (Sigma), for 1 h at room temperature. Alternatively, for von Kossa staining, sections were incubated in a 1% silver nitrate under ultraviolet light for 20 min, rinsed, immersed in 5% sodium thiosulfate for 5 min to remove unreacted silver, and counterstained with nuclear fast red for 5 min. Finally, specimens were dehydrated, cleared, and mounted. The samples were analyzed in a Zeiss Axiolab5 microscope and Axiocam208c imaging system (Zeiss). Histomorphometric data were measured on ImageJ software (version 1.51j8), which was calculated as a proportion of the total diaphysis’ area based on color thresholds. 

### 4.5. Gene Expression Analysis

Frozen femurs were powdered with a pestle and mortar in the presence of liquid nitrogen. Total RNA was isolated from DNA and proteins with Trizol^®^ (Invitrogen, San Diego, CA, USA) and chloroform, according to the established manufacturer’s protocol. The concentration and purity of total RNA were assessed by UV spectrophotometry (A260/A280) in a NanoDrop^®^ ND-1000 UV-Vis Spectrophotometer. RNA was reverse transcribed into complementary DNA (cDNA) with a two-step reverse transcription quantitative PCR Kit (iScript™ BioRad^®^), in accordance to the manufacturer's instruction. Following, quantitative PCR analysis was conducted in a Bio-Rad iQ5 real-time PCR system (Bio-Rad^®^) using SYBR Premix Ex Taq kit (Takara^®^). Optimized primers for amplification were acquired from BioRad: GAPDH (Unique Assay ID: qGgaCED0029996), ACE (Unique Assay ID: qGgaCED0024430), MAS1 (Unique Assay ID: qGgaCED0024192), AGTR1 (Unique Assay ID: qGgaCED0022835), and AGTR2 (Unique Assay ID: qGgaCED0023871). The relative gene expression level was normalized to the internal control (GAPDH) based on the 2^−ΔΔCt^ method. 

### 4.6. Statistical Analysis

Four independent experiences were conducted. The length of the all femur and diaphyseal mineralized region was measured three times. Regarding quantitative data, measurements were calculated and presented as mean ± standard deviation. Data normality was determined by the Shapiro–Wilk test. For normal datasets, one-way ANOVA was performed, followed by multiple comparisons using Tukey’s test. For non-parametric data sets, the Kruskal–Wallis test was performed, followed by multiple comparisons using Dunn’s tests. SPSS Statistics (IBM, version 26) was used for calculations. Statistical differences were considered to be significant if *p* values ≤ 0.05.

## 5. Conclusions

Bone formation in the embryonic femur is highly responsive to the negative effects of AngII, as well as to the preventive effects of AngII-receptor blockade. This model seems particularly suitable to investigate the mechanisms underlying AngII/AT1R/AT2R activation and modulation, as well as an effective tool for drug screening.

## Data Availability

The data presented in this study are available on request from the corresponding author.
